# L-Carnitine Protection Against Cisplatin Nephrotoxicity In Rats: Comparison with Amifostin Using Quantitative Renal Tc 99m DMSA Uptake

**DOI:** 10.4274/MIRT.20.01

**Published:** 2011-04-01

**Authors:** Yakup Yürekli, Perihan Ünak, Çiğdem Yenisey, Türkan Ertay, Fazilet Zumrut Biber Müftüler, Emin İlker Medine

**Affiliations:** 1 Adnan Menderes University, Department of Nuclear Medicine, School of Medicine, Aydin, Turkey; 2 Ege University, Department of Nuclear Applications, Institute of Nuclear Sciences, Izmir, Turkey; 3 Adnan Menderes University, Department of Biochemistry, School of Medicine, Aydin, Turkey; 4 Dokuz Eylul University, Department of Nuclear Medicine, School of Medicine, Izmir, Turkey ü

**Keywords:** Spermatic cord torsion; radionuclide imaging; cryptorchidism; inguinal hernia

## Abstract

**Objective:** In this study, we aimed to investigate the cytoprotective effect of L-carnitine against cisplatin-induced nephrotoxicity and to compare its efficacy with that of amifostin by quantitative renal Tc 99m DMSA uptake.

**Material and Methods:** Male Wistar rats were randomly divided into six groups of six animals each. 1) Control (saline; 5 ml/kg intraperitoneally); 2) L-carnitine (CAR; 300 mg/kg intraperitoneally); 3) Amifostine (AMI; 200 mg /kg intraperitoneally); 4) Cisplatin (CIS;7 mg/kg intraperitoneally); 5) Cisplatin plus L-carnitine (CIS + CAR); 6) Cisplatin plus amifostine (CIS + AMI). L-carnitine and amifostine were injected 30 minutes before cisplatin in Group 5 and 6. Tc 99m DMSA, 7.4 MBq/0.2 ml, was injected through the tail vein 72 hours after the drug administration. Rats were killed and kidneys removed by dissection 2 hours after the injection of the radiopharmaceutical. The percentage of the injected dose per gram of kidney tissue (%ID/g) was calculated. Renal function was monitored by measuring BUN and plasma levels of creatinine. Lipid peroxidation and glutathione content were determined by measuring malondialdehyde (MDA) and reduced glutathione (GSH) in kidney tissue homogenates.

**Results:** Tc 99m DMSA uptake per gram tissue of the kidney as %ID/g was 29.54±4.72, 29.86 ± 7.47 and 26.37 ± 4.54 in the control, CAR and AMI groups respectively. %ID/g was the lowest of all the groups, 11.60±3.59 (p<0.01), in the cisplatin group. Carnitine or amifostine administration 30 minutes before cisplatin injection resulted a significant increase in %ID/g, 21.28±7.73 and 18.97±3.24 respectively, compared to those of cisplatin-treated rats (p<0.002). A marked increase in plasma BUN and creatinine indicating nephrotoxicity and acute renal failure was observed in the cisplatin-treated group. MDA and GSH levels were concordant with cisplatin-induced oxidative stress in the kidney tissue.

**Conclusion:** The results showed that L-carnitine significantly attenuates the cisplatin-induced nephrotoxicity as amifostin.

**Conflict of interest:**None declared.

## INTRODUCTION

Cisplatin is an antineoplastic agent in the treatment of many types of human tumors including testicular, head and neck, ovarian, cervical, nonsmall cell lung carcinoma. Its cytotoxic activity is mediated through DNA inter and intrastrand cross linking and adduct formation resulting in defective DNA templates and arrest of DNA synthesis and replication (1) Cisplatin induces significant ototoxicity, neurotoxicity and nephrotoxicity. The side effects of cisplatin, particularly dose-related nephrotoxicity, have been the major factor that restricts its optimal use in cancer therapy. Nephrotoxicity was observed in almost one-third of patients undergoing cisplatin treatment. Although the precise molecular mechanism of cisplatin nephrotoxicity is not known, recent research reveals that it is a complex multifactorial process involving necrosis, apoptosis, inflammation, ischemia and oxidative stress. It is known that renal tubules are the major sites of damage resulting in acute renal failure. Numerous agents have been tested to prevent cisplatin-induced nephrotoxicity (2,3,4) However, effective methods for clinical use has not been established yet. 

L-carnitine is a low-molecular-weight, natural compound obtained from the diet and also biosynthesized in vivo from the essential amino acids lysine and methionine. It is a cofactor required for transport of long-chain fatty acids into the mitochondrial matrix for energy production. However, biological functions of L-carnitine go far beyond its role in the transport of fatty acids. Carnitine and its acetylated derivatives facilitate the b-oxidation and improve energy metabolism, minimize the toxic effects of free forms of long-chain fatty acids in and around mitochondria, protect the mitochondrial membranes and prevent permeability transitions, therefore, supresses the release of free electrons that generate free radicals and cytochrome c that activates caspases. 

L-carnitine protects against oxidative stress and reduces the apoptosis rate (5,6,7,8,9).

Amifostine (Ethyol), a pro-drug that forms an activated free thiol through dephosphorylation by membrane-bound alkaline phosphatase in normal cells. AMI selectively protects normal cells from toxic effects of antineoplastic agents by scavenging free radicals, donating hydrogen ions, binding to active metabolites antineoplastic agents. There is a growing amount of evidence of its ability to selectively protect almost all normal tissues except central nervous system, but not neoplastic tissues from the cytotoxic effects of some of chemotherapeutic agents and radiation therapy. Amifostine was recommended in the American Society of Clinical Oncology Clinical Practice Guidelines for the Use of Chemotherapy and Radiotherapy Protectants for the prevention of cisplatin-related toxicity (10,11,12,13) Tc 99m DMSA, meso-2,3-dimercaptosuccinic acid, is used for functional imaging of the proximal renal tubular mass. It is accumulated in the cytoplasm of the proximal tubular cells by peritubular uptake which is mediated by the organic anion transport system. Its uptake depends on the renal blood flow and proximal tubular cell membrane transport function (14,15,16). Tc 99m DMSA scintigraphy has been used clinically to diagnose and monitor ifosfamid-induced tubular dysfunction (17) and cisplatin and cisplatin/ifosfamid nephrotoxicity in children (18).

Because, oxidative stress has been recognized as a major factor in the mechanism of cisplatin nephrotoxicity, lipid peroxidation and glutathione content were determined by measuring malondialdehyde and reduced glutathione in kidney tissue homogenates.

In this study, we aimed to investigate the role of L-carnitine in the protection of cisplatin-induced nephrotoxicity and to compare its efficacy with that of cytoprotective agent amifostin by using quantitative Tc 99m DMSA uptake measurements and biochemical parameters. 

## MATERIALS AND METHODS

Male Wistar albino rats, weighing 190-260 g, were provided by the Experimental Surgery and Research Laboratories of Ege University. 

Rats were housed in standard laboratory conditions and 12-hour light-dark cycle and had free access to commercial food and tap water. The study protocol was approved by the Institutional Animal Review Committee of Ege University (Ref no: 2005-04) and all animals have received human care in compliance with the Guide for the Care and Use of Laboratory Animals.Rats were randomly divided into six groups. Each group consisted of six rats. 1) Control (saline; 5 ml/kg intraperitoneally); 2) L-carnitine (CAR; 300 mg/kg intraperitoneally); 3) Amifostine (AMI; 200 mg /kg intraperitoneally); 4) 

Cisplatin (CIS; 7 mg/kg intraperitoneally); 5) Cisplatin plus L-carnitine (CIS + CAR); 6) Cisplatin plus amifostine (CIS + AMI). (Table 1). Amifostine and L-carnitine were injected 30 minutes before cisplatin in Group 5 and 6. Cisplatin dose is determined according to the cisplatin model of acute renal failure in rats described previously (19,20). Rats were killed by heart puncture under intense ether atmosphere 72 hours after the injections. Cisplatin (DBL, 10 mg/10ml vial, FH Faulding & Co Limited Australia), amifostine (Ethyol, 500 mg, TR Erkim Ltd Sti) and l-carnitine (Carnitene, 1 g, Sigma Tau, Italy) were purchased commercially. Amifostine was dissolved in 9.7 ml of double distilled water. 

Tc 99m, as sodium pertechnetate, was eluted from a 99Mo/Tc 99m generator (Monrol A.Ş. Istanbul, Turkey) just before the radiolabelling procedure. DMSA (TechneScan, Mallinckrodt Medical B.V Holland) was purchased as a freeze-dried commercial kit containing 1.2 mg dimercaptosuccinic acid, 0.3 mg stannous chloride dehydrate and 30 mg inositol. Tc 99m DMSA was prepared by adding 740 MBq of Tc 99m pertechnetate to 3 ml of saline to the kit. Quality control procedures were performed according to the manufacturer’s instructions after 15 minutes’ incubation at room temperature. Labeling efficiency was greater than 95%.

**Quantitative Tc 99m DMSA Uptake **

Tc 99m DMSA, 7.4 MBq/0.2 ml, was administered through the tail vein. Rats were killed and the kidneys were removed by dissection 2 hours after the radiopharmaceutical injection. Samples of kidneys were weighed in pre-weighed containers. Radioactivity in each organ sample was counted using a Cd (Te) detector equipped with a RAD 501 single-channel analyzer. The activity was expressed as a percentage of the injected dose per gram of tissue (%ID/g). The %ID/g activity was calculated by dividing the activity in each kidney by total activity injected and the mass of the organ.

**Biochemical Assays**

The blood samples from the treatment groups were collected in heparinized vials and centrifuged at 3500 rpm for 15 min to isolate serum. BUN and creatinine levels in serum were measured with an autoanalyser using commercial diagnostic kits (Sigma-Aldrich Chemie, Germany). One of the kidneys of each animal was washed with cold saline and kept in deep freezer (-80 ºC) until homogenization. The homogenates were centrifuged at 3500 rpm and supernatants were collected and used for the determination of malondialdehyde (MDA) and reduced glutathione (GSH). MDA level was determined by a method based on the reaction with thiobarbituric acid (TBA) at 90–100 °C (21). GSH content in tissue supernatants was measured according to the method of Beutler et al (22). 

**Statistical Analysis**

Data are expressed as the mean±SD. The nonparametric Kruskal-Wallis test was performed on the data of radiopharmaceutical uptake and biochemical variables to examine the differences among groups. Mann-Whitney U test was used to compare two in dependent samples. Statistical differences were considered significant when P value was smaller than 0.05 with two sided probability.

## RESULTS

Intraperitoneal injection of cisplatin at a single dose of 7 mg/kg body weight resulted in acute renal failure as indicated by significant increase in plasma BUN and creatinine levels compared with controls (p<0.001). L-carnitine and amifostine administered 30 minutes before cisplatin prevented the cisplatin-induced elevations in BUN and creatinine. Cisplatin induced a significant increase in the MDA content of the kidneys and caused depletion of GSH as compared to the control group (p< 0.01). No significant changes were observed in MDA and GSH when carnitine or amifostin was administered prior to cisplatin (Table 2).

**Figure 1:**
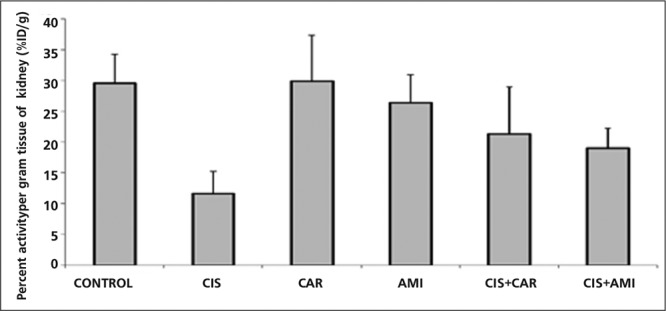
The percentage of the injected Tc 99m DMSA dose per gramof kidney tissue (%ID/g) in Control, CIS, CAR, AMI, CIS+CAR andCIS+AMI groups are shown.

The percentage of the injected dose per gram of kidney tissue in Control, CIS, CAR, AMI, CAR+CIS and AMI+CIS groups are shown in Fig 1. Tc 99m DMSA uptake per gram tissue of the kidney as %ID/g was 29.54±4.72, 29.86±7.47 and 26.37±4.54 in the control, CAR and AMI groups respectively (p>0.05). Cisplatin led to a significant decrease in tubular uptake of Tc 99m DMSA. %ID/g was the lowest of all the groups, 11.60±3.59 (p<0.01), in the cisplatin group. Carnitine or amifostine administration 30 minutes before cisplatin injection resulted a significant increase in the uptake of DMSA as %ID/g (21.28 ± 7.73 and 18.97±3.24 respectively) compared to those of cisplatin-treated rats (p<0.002). There was no statistically significant difference between the CAR only and AMI only groups (Figure 1).

## DISCUSSION

Nephrotoxicity prevents the application of appropriate therapeutic schedules with higher doses of cisplatin and limits its treatment success significantly. Renoprotective agents have been studied extensively because they are important in terms of preventing cisplatin-induced nephrotoxicity and improving its therapeutic potential. Numerous agents have been tested for renoprotection against cisplatin toxicity (3). 

L-carnitine is a natural compound whose biological functions go far beyond its role in the transport of fatty acids. Carnitine inhibits oxidative stress, apoptosis and inflammation (5). There are many experimental studies reporting the protective effect of carnitine against the toxicity of irradiation and antineoplastic agents including doxorubicin and cisplatin (23,24,25,26,27,28,29,30,31,32). In the studies investigating the potential renoprotective effect of carnitine against cisplatin-induced nephrotoxicity, it has been demonstrated that carnitine successfully prevented renal dysfunction and injury (23,24,25) But, a recent study concludes that neither carnitine nor amifostin has protective effect against cisplatin induced nephrotoxicity (33). Carnitine doses of 100-500 mg/kg were used in various types of experimental studies (23). We used an average dose within this range.

In the present study, intraperitoneal administration of a single dose of 7 mg/kg cisplatin resulted in acute renal failure as indicated by the increased BUN and serum creatinine measured at 72 hour. Oxidative stress and lipid peroxidation is strongly related to cisplatin-induced nephrotoxicity (34,35) Malondialdehyde is an indicator of lipid peroxidation and oxidative stress. Glutathione is a marker of antioxidant status. 

Cisplatin induced significant elevation in the renal tissue MDA and decreased the GH content of the rat kidneys compared to the controls. Both L-carnitine and amifostine significantly inhibited MDA elevation and GH depletion induced by cisplatin (p<0.05).Cisplatin induced a significant decrease in the uptake of Tc 99m DMSA in the rats’ kidneys compared to the control group (p<0.001) consistent with tubular injury and nephrotoxicity. Renal function was monitored by measuring BUN and plasma levels of creatinine. In the groups which rats were pretreated with 300 mg/kg L-carnitine or 200 mg/kg amifostine before cisplatin injection, no significant decrease in the uptake of Tc 99m DMSA was observed, which is concordant with the protective effect of both L-carnitine and amifostine. Although there is no significant difference between the renal DMSA %ID/g of CIS+CAR and CIS+AMI groups, L-carnitine seems to have higher protective effect than amifostine. Our results confirmed the previous experimental studies indicating the protective effect of L-carnitine against cisplatin-induced toxicity. 

Because, scintigraphic imaging with measurement of renal activity as counts per pixel is nearly equevalent of this quantitative technique, it is possible to determine the grade of renal toxicity and the potential protective effect of the agents tested, to compare different agents and combinations simply and accurately without sacrificing animals.

The methods for the evaluation of cisplatin-induced nephrotoxicity in experimental studies are based on the measurements of biochemical markers of renal function and indicators of oxidative stress and histopathological findings of renal injury. In this study, we demostrated the cisplatin nephrotoxicity and the protective effect of L-carnitine by using quantitative renal Tc 99m DMSA uptake. 

## Figures and Tables

**Table 1:**
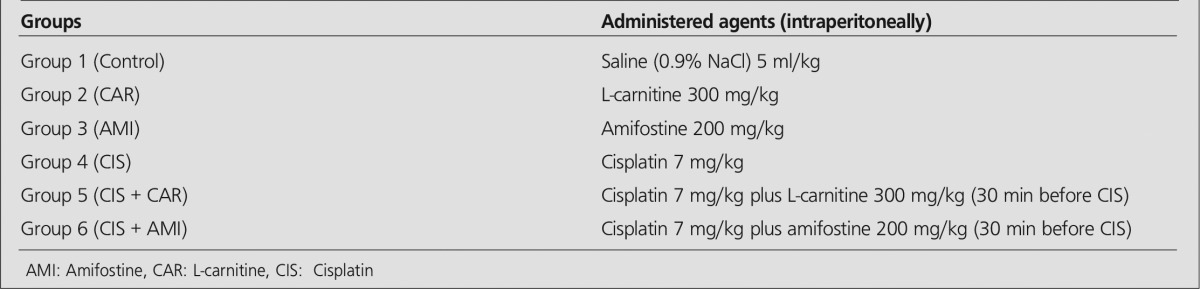
Study groups and experimental design

**Table 2:**
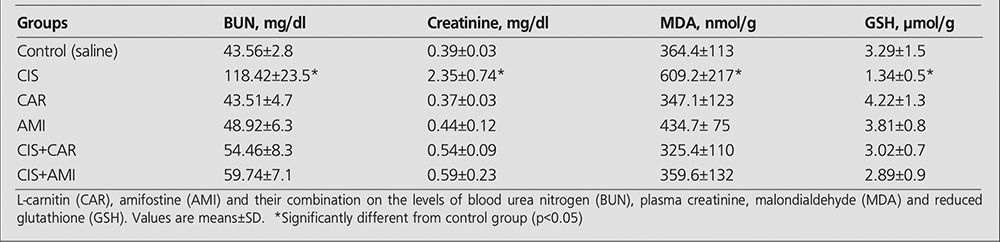
Effect of cisplatin (CIS)

## References

[1] Cohen SM, Lippard SJ (2001). Cisplatin: from DNA damage to cancer chemotherapy. Prog Nucleic Acid Res Mol Biol.

[2] Yao X, Panichpisal K, Kurtzman N, Nugent K (2007). Nugent K. Cisplatin nephrotoxicity: a review. Am J Med Sci.

[3] Pabla N, Dong Z (2008). Cisplatin nephrotoxicity:mechanisms and renoprotective strategies. Kidney Int.

[4] Kintzel PE (2001). Anticancer drug-induced kidney disorders. Drug Saf.

[5] Chapela SP, Kriguer N, Fernandez E.H, Stella CA (2009). Involvement of L-Carnitine in Cellular Metabolism: Beyond Acyl-CoA Transport. Mini-Reviews in Medicinal Chemistry.

[6] Martelli E, Caso V (2001). Carnitine protects mitochondria and removes toxic acyls from xenobiotics. Drugs Exp Clin Res.

[7] Ghelardini C, Calvani M, Nicolai R, Mosconi L, Vivoli E, Pacini A, Bartolini A (2007). Protective effect of acetyl- L –carnitine on the apoptotic pathway of peripheral neuropathy. Eur J Neurosci.

[8] Ghelardini C, Calvani M, Nicolai R, Mosconi L, Toscano A, Pacini A, Bartolini A (2009). Neuroprotective effects of acetyl- L -carnitine on neuropathic pain and apoptosis: a role for the nicotinic receptor. J Neurosci Res.

[9] Zhu X, Sato EF, Wang Y, Nakamura H, Yodoi J, Inoue M (2008). Acetyl- L -carnitine suppresses apoptosis of thioredoxin 2-deficient DT40 cells. Arch Biochem Biophys.

[10] Mahaney Jr FX (1990). Agent designed for war now in doctor’s armamentarium. J Natl Cancer Inst.

[11] Koukourakis MI (2002). Amifostine in clinical oncology: current use and future applications. Anticancer Drugs.

[12] Culy CR, Spencer CM (2001). Amifostine: An update on its clinical status as a cytoprotectant in patients with cancer receiving chemotherapy or radiotherapy and its potential therapeutic application in myelodysplastic syndrome. Drugs.

[13] Hensley ML, Hagerty KL, Kewalramani T, Green DM, Meropol NJ, Wasserman TH, Cohen GI, Emami B, Gradishar WJ, Mitchell RB, Thigpen JT, Trotti A 3rd, von Hoff D, Schuchter LM (2009). American Society of Clinical Oncology 2008 clinical practice guideline update: use of chemotherapy and radiation therapy protectants. J Clin Oncol.

[14] Muller-Suur R, Gutsche HU (1995). Tubular reabsorption of technetium-99m DMSA. J Nucl Med.

[15] van Luyk, GJ Ensing, DA Piers (1983). Low renal uptake of Tc 99m DMSA in patients with proximal tubular dysfunction. Eur J Nucl Med.

[16] Provoost AP, Van Aken M (1985). Renal handling of technetium-99m DMSA in rats with proximal tubular dysfunction. J Nucl Med.

[17] Caglar M, Yaris N, Akyuz C (2001). The utility of (99m)Tc-DMSA and Tc(99m) EC scintigraphy for early diagnosis of ifosfamide induced nephrotoxicity. Nucl Med Comm.

[18] Anninga JK, Valdes Olmos RA, Kraker J, Tinteren H, Hoefnagel CA, Royen EA (1994). Technetium-99m dimercaptosuccinic acid and ifosfamide tubular dysfunction in children with cancer. Eur J Nucl Med.

[19] Zhang JG, Viale M, Esposito M, Lindup WE (1999). Tiopronin protects against the nephrotoxicity of cisplatin in the rat. Hum Exp Toxicol.

[20] Yildirim Z, Sogut S, Odaci E, Iraz M, Ozyurt H, Kotuk M, Akyol O (2003). Oral erdosteine administration attenuates cisplatin-induced renal tubular damage in rats. Pharmacol Res.

[21] Wasowicz W, Neve J, Peretz A (1993). Optimized steps in fuorometric determination of thiobarbituric acid-reactive substances in serum: Importance of extraction pH and infuence of sample preservation and storage. Clin Chem.

[22] Beutler E, Durgun O, Kelly BM (1963). Improved method for the determination of blood glutathione. J Lab Clin Med.

[23] Chang B, Nishikawa M, Sato E, Utsumi K, Inoue M (2002). L-Carnitine inhibits cisplatin-induced injury of the kidney and small intestine. Arch Biochem Biophys.

[24] Sayed-Ahmed MM, Eissa MA, Kenawy SA, Mostafa N, Calvani M, Osman AM (2004). Progression of cisplatin-induced nephrotoxicity in a carnitine-depleted rat model. Chemotherapy.

[25] Tufekci O, Gunes D, Ozogul C, Kolatan E, Altun Z, Yilmaz O, Aktas S, Erbayraktar Z, Kirkim G, Mutafoglu K, Soylu A, Serbetcioglu B, Guneri EA, Olgun N (2009). Evaluation of the effect of acetyl L-carnitine on experimental cisplatin nephrotoxicity. Chemotherapy.

[26] Boonsanit D, Kanchanapangka S, Buranakarl C (2006). L-carnitine ameliorates doxorubicin-induced nephrotic syndrome in rats. Nephrology (Carlton).

[27] Al-Majed AA, Sayed-Ahmed MM, Al-Yahya AA, Aleisa AM, Al-Rejaie SS, Al-Shabanah OA (2006). Propionyl-L-carnitine prevents the progression of cisplatin-induced cardiomyopathy in a carnitine-depleted rat model. Pharmacol Res.

[28] Pisano C, Pratesi G, Laccabue D, Zunino F, Lo Giudice P, Bellucci A, Pacifici L, Camerini B, Vesci L, Castorina M, Cicuzza S, Tredici G, Marmiroli P, Nicolini G, Galbiati S, Calvani M, Carminati P, Cavaletti G (2003). Paclitaxel and Cisplatin-induced neurotoxicity: a protective role of acetyl-L-carnitine. Clin Cancer Res.

[29] Sayed-Ahmed MM, Salman TM, Gaballah HE, Abou El-Naga SA, Nicolai R, Calvani M (2001). Propionyl-L-carnitine as protector against adriamycin-induced cardiomyopathy. Pharmacol Res.

[30] Andrieu-Abadie N, Jaffrezou JP, Hatem S, Laurent G, Levade T, Mercadier JJ (1999). L-carnitine prevents doxorubicin-induced apoptosis of cardiac myocytes: role of inhibition of ceramide generation. FASEB J.

[31] Luo X, Reichetzer B, Trines J, Benson LN, Lehotay DC (1999). L-carnitine attenuates doxorubicin-induced lipid peroxidation in rats. Free Radic Biol Med.

[32] Caloglu M, Yurut-Caloglu V, Durmus-Altun G, Oz-Puyan F, Ustun F, Cosar-Alas R, Saynak M, Parlar S, Turan FN, Uzal C (2009). Histopathological and scintigraphic comparisons of the protective effects of L-carnitine and amifostine against radiation-induced late renal toxicity in rats. Clin Exp Pharmacol Physiol.

[33] Uzunoglu S, Karagol H, Ozpuyan F, Cosar R, Cicin I, Yurutcaloglu V, Denizli B, Tanriverdi O, Sut N (2011). Protective effect of L-carnitine versus amifostine against cisplatin-induced nephrotoxicity in rats. Med Oncol.

[34] Vermeulen NP, Baldew GS (1992). The role of lipid peroxidation in the nephrotoxicity of cisplatin. Biochem Pharmacol.

[35] Nakano S, Gemba M (1989). Potentiation of cisplatin-induced lipid peroxidation in kidney cortical slices by glutathione depletion. Jpn Pharmacol.

